# The resistance of intracellular mediators to doxorubicin and cisplatin are distinct in 3D and 2D endometrial cancer

**DOI:** 10.1186/1479-5876-10-38

**Published:** 2012-03-06

**Authors:** Kenny Chitcholtan, Peter H Sykes, John J Evans

**Affiliations:** 1 Department of Obstetrics and Gynaecology, University of Otago, 2 Riccarton Avenue, Christchurch 8011, New Zealand; 2 Department of Obstetrics and Gynaecology, Christchurch Women's Hospital, 2 Riccarton Avenue, Christchurch 8011, New Zealand; 3 Department of Obstetrics and Gynaecology, Centre of Neuroendocrinology and MacDiarmid Institute of Advanced Materials and Nanotechnology, University of Otago, 2 Riccarton Avenue, Christchurch 8011, New Zealand

## Abstract

**Background:**

Advanced endometrial cancer often shows resistance to clinical chemotherapy although potencies of anticancer drugs *in vitro *are promising. The disparity suggests that *in vi*vo microenvironments are not recapitulated by *in vitro *models used for preclinical testing. However, spheroids replicate some important properties of tumours *in vivo*. Therefore, for the first time, we compared effects of doxorubicin and cisplatin on 3D multicellular structures and 2D cell monolayers of endometrial cancer cells.

**Methods:**

3D multicellular structures were generated by culturing cancer cells on non-adherent surfaces; and for comparison cell monolayers were cultured on adherent culture plates. Ishikawa, RL95-2, and KLE cell lines were studied. Morphologies of 3D multicellular structures were examined. After 48 hours treatment with anticancer drugs, apoptosis, proliferation, glucose metabolism and vascular endothelial growth factor (VEGF) were analysed. Immunostaining of PCNA, Glut-1, p-Erk1/2, SOD-1 and p-Akt1/2/3 was also performed.

**Results:**

Distinct 3D multicellular morphologies were formed by three different endometrial cancer cell lines. Doxorubicin induced less apoptosis in 3D multicellular structures of high grade cancer cells (RL95-2 and KLE cell lines) than in cell monolayers. Parallel alterations in Erk1/2 phosphorylation and cell proliferation might suggest they were linked and again doxorubicin had less effect on 3D multicellular structures than cell monolayers. On the other hand, there was no correlation between altered glucose metabolism and proliferation. The responses depended on cancer cell lines and were apparently not mediated by altered Glut-1 levels. The level of SOD-1 was high in 3D cell cultures. The effects on VEGF secretion were various and cancer cell line dependent. Importantly, both doxorubicin and cisplatin had selective paradoxical stimulatory effects on VEGF secretion. The microenvironment within 3D multicellular structures sustained Akt phosphorylation, consistent with it having a role in anchorage-independent pathways.

**Conclusions:**

The cancer cells responded to microenvironments in a distinctive manner. 3D multicellular structures exhibited greater resistance to the agents than 2D monolayers, and the differences between the culture formats were dependent on cancer cell lines. The effects of anticancer drugs on the intracellular mediators were not similar in 3D and 2D cultures. Therefore, using 3D cell models may have a significant impact on conclusions derived from screening drugs for endometrial carcinomas.

## Background

Endometrial carcinoma is the most common gynaecologic malignancy in developed countries [1-3]. The early stage of disease is highly curable with a 5-year survival rate of more than 80% [4]. However, advanced disease has low survival rates of less than 20%, and metastatic appearance is a significant cause of mortality [5]. Chemotherapeutic regimens for endometrial cancer include doxorubicin and cisplatin. Doxorubicin increases cell death through multiples pathways [6]. Cisplatin is a platinum-based drug and is believed to affect proliferation and apoptosis [7,8]. Only 20-25% of patients respond to these agents suggesting the efficacy of chemotherapy in the clinic is less effective than results obtained from evaluation of *in vitro *2D cell culture models [9]. Therefore, a cell model, which represents physiological behaviours of tumour, is urgently needed for studying endometrial cancer.

In recent years, 3D multicellular structures, sometimes called spheroids, have gained attention for their use in screening novel anticancer drugs. Numerous experimental data *in vitro *have suggested that spheroids represent physiological tumours better than cell monolayers [10,11]. The behaviour and growth of cancer cells in spheroids have been studied to a limited extent for some solid tumours including breast, colon, prostate, and ovarian tumours but not at all for endometrial cancer [12-16]. Spheroids of cancer cells are potentially valuable cell models for studying tumour growth and development prior to establishment of angiogenesis and during the metastatic process [14]. Spheroids are composed of heterogeneous cancer cell populations that have distinct energy and nutrient metabolism, and complex cell-cell and cell-extracellular matrix interactions [10,11,17]. The responses of anticancer agents in spheroids may more closely reflect the true efficacy of agents observed in clinical settings.

The advantages of using multicellular structures over cell monolayers have been suggested. However, there is no data on the use of multicellular structures for studying the behaviour of endometrial cancer. We hypothesised that multicellular structures of endometrial cancer might exhibit greater resistance to doxorubicin and cisplatin than cell monolayers and portray the *in vivo *response more accurately. Therefore, the purpose of this work was, for the first time, to investigate and compare antitumour activities of doxorubicin and cisplatin in multicellular structures and cell monolayers of endometrial cancer cell lines. In this study, we use 'spheroid' to mean a multicellular structure that has a compact structure and the diameter is greater than 100 μm. The endpoint analysis after drug treatments included apoptosis, proliferation markers, glucose metabolism markers, endogenous antioxidant protein, vascular endothelial growth factor (VEGF) secretion and expression of the intracellular mediators, Akt, Erk and their phosphorylated forms. Some of these biomarkers are used in the clinical prognostic evaluation after anticancer drug treatment.

## Materials and methods

### Cell lines and reagents

Endometrial cancer cell lines Ishikawa (grade 1 with *PTEN *and *p53 *mutations) was gifted by Dr Masato Nishida, Kasumigaura National Hospital, Tsuchiura-shi, Ibaraki-ken, Japan. RL95-2 (grade 2, with *PTEN *and *p53 *mutations) was purchased from ATCC. KLE (grade 3, with wild type *PTEN *but *p53 *mutation), was gifted by Professor Eric Asselin, University of Quebec at Trois-Rivieres, Quebec, Canada. Ishikawa and RL95-2 cells were maintained in MEM medium (GIBCO^®^, Invitrogen, New Zealand) supplemented with 10% fetal bovine serum (FBS), 500 units/ml of penicillin/streptomycin and 1 mM glutamax. KLE cells were maintained in DMEF-F12 medium (GIBCO^®^, Invitrogen) supplemented with 10% FBS, 500 units/ml of penicillin/streptomycin and 1 mM glutamax. Doxorubicin, cisplatin, bromodeoxyuridine (BrdU) and propidium iodide (PI) were purchased from Sigma-Aldrich (New Zealand). Antibodies (Erk1/2, BrdU, p-ERK1/2, Akt, p-Akt (Ser-473), GAPDH, SOD1, β-actin, β1-integrin, anti-Mouse IgG-HRP, and anti-Rabbit IgG-HRP) were purchased from Santa Cruz Biotechnology (CA, USA).

### Cell culture

Generation of multicellular structures: twenty-four well culture plates were coated with poly-HEMA at 37°C overnight with continuous shaking. Prior to cell culture, culture well plates were washed once with PBS pH 7.4. The cells were plated in 24-well plates at a density of 100,000 cells/well. For monitoring the growth of cells in 3D multicellular structures, cells were collected and incubated with trypsin-EDTA for 10-20 minutes prior to counting them with a haemocytometer. For cell monolayers, cells were plated at a density 100,000 of cells/well. Cells were incubated at 37°C in a humidified 5% CO_2 _atmosphere for 5 days.

### Determination the compactness of a 3D multicellular structure

After 5 days of culture, spheroids, cell aggregates and cell clusters were incubated with trypsin-EDTA for 7 minutes and triturated with 1 ml pipette. The enzymatic reaction was then terminated by addition of PBS. Differential interface contrast (DIC) images were captured with epifluorescence microscopy (AxioVision 4.5. Apotome software, Carl Zeiss, Oberkochen, Germany).

### Treatment with clinical drugs

After 5 days culturing, the supernatants were replaced with 1 ml fresh medium. Agents were added to cells and incubated for a further 48 hours. Doxorubicin and cisplatin were dissolved in 100% DMSO, and a similar amount of DMSO was added in the control.

### Detection of cell apoptosis using Annexin-V/Propidium iodide (PI)

After treatment with potential agents, cells were harvested, trypsinised, washed and centrifuged. Cell pellets were resuspended in binding assay buffer and annexin-V conjugated FITC solution (Invitrogen, New Zealand) was added. Cells were then incubated in the dark at room temperature for 20 minutes. Propidium iodide (PI) was then added at final concentration of 10 μg/ml. Annexin-V positive cells were analysed by FACS (Beckman Coulter). Data were collected from at least four independent experiments and were then analysed with CXP Software (Beckman Coulter).

### Measurement of cell proliferation by BrdU incorporation

After cells were treated with agents, BrdU at final concentration at 20 μM was added and incubated for a further 5 hours at 37°C in a 5% CO_2 _atmosphere. Cells were harvested, trypsinised and fixed with 4% paraformaldehyde in PBS pH 7.4 and then washed with PBS pH 7.4. Cells were permeabilised with 0.1% Triton X-100 for 20 minutes and washed. Cells were incubated with anti-BrdU antibody overnight at 4°C, washed and stained with anti-mouse IgG-FITC for 60 minutes and further incubated with 10 μg/ml PI for 20 minutes. Cells were then analysed by FACS (Beckman Coulter) and data were collected from at least four independent experiments and were then analysed with CXP Software, Beckman Coulter).

### Measurement of glucose metabolism by uptake of 2-[N-(7-nitrobenz-2-oxa-1,3-diazol-4-yl)amino]-2-deoxy-D-glucose (2-NBDG)

Multicellular structures were washed once with PBS pH 7.4 and then were suspended in 1 ml assay buffer and 2-NBDG was added at 20 μM final concentrations. Cells were incubated at 37°C in a humidified 5% CO_2 _atmosphere for 60 minutes and were washed with ice cold PBS pH 7.4 and were trypsinised. Cell suspensions were kept in cold assay buffer and 2-NBDG stained cells were analysed with FACS (Beckman Coulter) and data were collected from at least four independent experiments and were then analysed with CXP Software (Beckman Coulter). For cell monolayers, cells were first trypsinised prior to incubation with 2-NBDG.

### Indirect immunofluorescent analysis

Multicellular structures were fixed with 4% paraformaldehyde in PBS pH 7.4 for 40 minutes. The 3D multicellular structures were washed and embedded in mixtures of OTC: PBS pH 7.4 (70%:30%). Frozen sections were cut 7 μm thick and placed on polylysine coated slides. The sections were blocked with 5% BSA in PBS pH 7.4 for 60 minutes and were washed with PBS pH 7.4. The cut sections were incubated with -20°C methanol (99%) for 10 minutes and washed with ice cold PBS pH 7.4 and then incubated with a 1/200 dilution of primary antibodies overnight at 4°C. The sections were then washed and incubated with a 1/500 dilution of secondary Alexa™ 488- or FITC-conjugated antibodies at 37°C for 60 minutes. The sections were stained with 10 μg/ml Hoechst (Molecular Probe, Invitrogen) at 37°C for 20 minutes. The sections were washed extensively with ice cold PBS pH 7.4 plus 0.05% Tween-20. Anti-fading (Dako Florescent Mounting Medium) was added and sections were analysed with epifluorescence microscopy (AxioVision 4.5. Apotome software, Carl Zeiss, Oberkochen, Germany). Fluorescent images were collected from at least two independent experiments and at least 7 images from each experiment were captured and analysed.

### Immunoblotting analysis

Multicellular structures and adherent cells were lysed with cold RIPA buffer containing protease inhibitor cocktail tablets (Complete Mini, Roche, New Zealand) on ice for 30 minutes. Sample buffer was added and protein lysate was boiled at 95°C for 5 minutes. Cells were centrifuged at 14,000 rpm at 4°C for 10 minutes. Proteins were loaded and separated with SDS-PAGE using 5% stacking and 7.5-10% separating gels. Proteins were then electro-transferred onto PVDF membranes (100 Voltage, 60 minutes). The membrane was blocked with 5% non-fat milk powder in TBS-T buffer for 60 minutes. Membranes were then washed and incubated with primary antibodies over night at 4°C. Membranes were washed with TBS-T, incubated with a secondary peroxidase-conjugated antibody for 90 minutes and washed. Antibody localisation was determined using an enhanced chemiluminescent detection system ECL (Amersham, GE Healthcare). To ensure equal protein loading GAPDH and beta-actin proteins were used as a house keeping protein. Cell lysate from at least four independent experiments were collected and analysed for western blotting. Protein bands were detected and analysed by using Alliance 4.7, Unitec (Cambridge, UK).

### ELISA of vascular endothelial growth factor (VEGF)

ELISA of VEGF was performed using the DuoSet Human VEGF ELISA Kit (R&D System) that detects VEGF-A isoforms. Cell media from at least four independent experiments were collected and analysed for VEGF.

### Statistical analysis

Statistics were performed using SigmaPlot 11. Data were statistically analysed using Student's *t*-test and ANOVA and *P *< 0.05 was considered significant. All data are presented as mean ± SEM.

## Results

### Different subtypes of endometrial cancer generated distinct morphologies of spheroids

**Figure 1:**
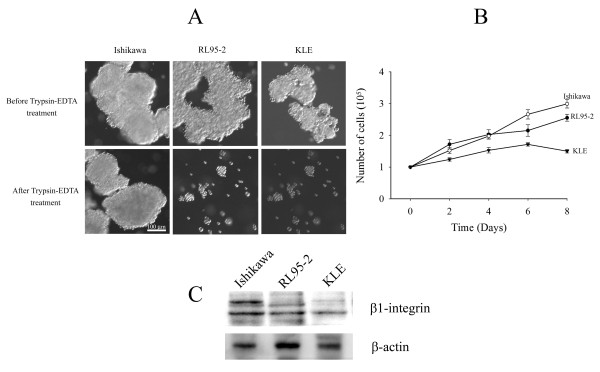
**Morphology, growth pattern, and expression of β1-integrin**. (A) Differential interface contrast (DIC) images of 3D multicellular morphologies from structures formed by epithelial endometrial cancer cell lines after 5 days cultured on poly-HEMA coated cultureware. Ishikawa cell line formed tightly compact spheroids, which were resistant to trypsin-EDTA digestion. RL95-2 and KLE cell lines formed cell aggregates and clusters respectively, and they were easily dissociated by trypsin-EDTA treatment. The scale bar is 100 μm. (B) The growth of Ishikawa, RL95-2 and KLE cell lines cultured in 3D structures. The total cell number was counted by a haemocytometer at intervals of 2 days. The open circle (○), dark circle (●) and triangle (▼) represent Ishikawa, RL95-2 and KLE cell lines, respectively. (C) The expression of β1-integrin was observed in Ishikawa, RL95-2 and KLE cell lines.

After 24 hours of culturing, small aggregations of cells were observed (data not shown), and larger multicellular structures formed after 5 days of culture (Figure 1A). Ishikawa cells formed large, tightly compact spheroids, which have defined margins and diameter greater than 100 μm. The compact spheroids were resistant to the enzymatic treatment of trypsin-EDTA. On the other hand, RL95-2 cells tended to form loose multicellular aggregates, which were easily dissociated by trypsin-EDTA digestion. KLE cells tended to develop small cell clusters that were dissociated into single cells after trypsin-EDTA treatment. The average diameter of 3D multicellular structures of Ishikawa, RL95-2 and KLE cells before the enzymatic treatment were 168.60 ± 9.18, 135.39 ± 8.5 and 43.72 ± 3.55 μm, respectively. The growth of multicellular structures was monitored by counting total cell numbers at intervals of 2 days (Figure 1B). Increasing cell numbers of Ishikawa and RL95-2 cell lines were correlated with increasing time of culture. However, KLE cell line reached a plateau after 6 days of culture. The formation of compact spheroids in Ishikawa cell line, cell aggregates in RL95-2 and cell clusters in KLE cell line was not associated with the expression of β1-integrin (Figure 1C).

### Effects of doxorubicin and cisplatin on viability and apoptosis

**Figure 2:**
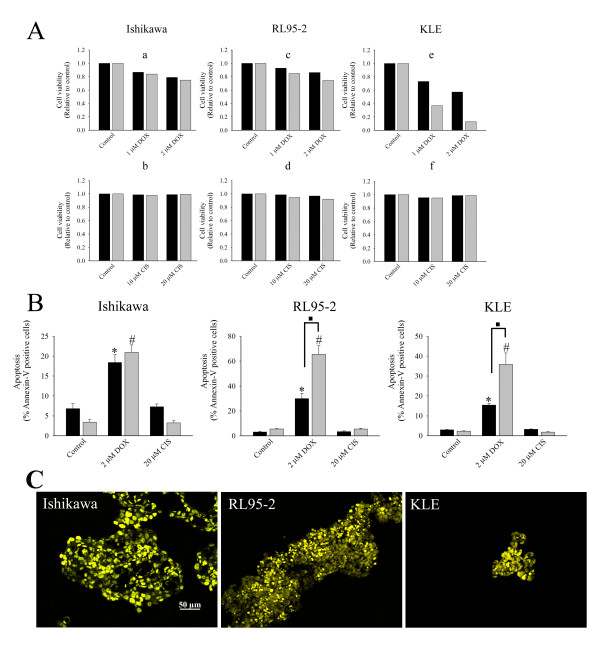
**Cell viability, apoptosis and distribution of doxorubicin (DOX)**. (A) Cell viability after 48 hours treatment with doxorubicin (DOX) and cisplatin (CIS). The treated cells were stained with PI and analysed with FACS. The PI positive cell staining was calculated relatively to the control. The (■) dark and (grey square symbol) grey bars represent multicellular structures and cell monolayers, respectively. (B) After 48 hours treated with anticancer agents, cells were stained with annexin-FITC and PI and analysed with FACS. Multicellular structures and cell monolayers of Ishikawa, RL95-2 and KLE cells were studied. The data are presented as mean ± SEM from at least four separate experiments. The (■) dark and (grey square symbol) grey bars represent 3D multicellular structures and cell monolayers respectively. (*) *P *< 0.05, compared to control multicellular structures. (#) *P *< 0.05, compared to control cell monolayers. (■) *P *< 0.05, comparison of between 3D cell cultures and cell monolayers. (C) Distribution of doxorubicin (DOX) within 3D multicellular structures of Ishikawa, RL95-2 and KLE after 48 hr incubations. DOX was located in the nucleus of cancer cells in multicellular structures. Images were obtained and representative of two independent experiments.

Doxorubicin reduced cell viability of Ishikawa, RL95-2 and KLE cell lines in a dose dependent manner (Figure 2A-a, c, e). However, 3D multicellular structures of these cell lines had higher viability than their cell monolayer counterparts. Cisplatin had limited effects on the reduction of cell viability of Ishikawa and KLE cell lines (Figure 2B-b, f) but it slightly decreased the viability of RL95-2 cell line in the 2D culture (Figure 2B-d).

Next, we determined whether reduction of cell viability was due to induction of apoptosis-related cell death. Doxorubicin increased the percentage of apoptotic cells in both spheroids and cell monolayers of Ishikawa cells (Figure 2B). Similarly, doxorubicin strongly induced apoptosis in RL95-2 cells. However, cell aggregates of RL95-2 cell line had significantly fewer apoptotic cells than cell monolayers. Doxorubicin also increased apoptosis in both cell clusters and monolayers of KLE cell line and again cell clusters were less sensitive to doxorubicin than cell monolayers. Cisplatin did not produce an increase of apoptotic cells in any cell lines. Immunofluorescent images showed doxorubicin was distributed throughout 3D multicellular structures of all cancer cell lines (Figure 2C). This would suggest that drug accessibility does not account for the insensitivity of cells in multicellular structures to doxorubicin.

### Effects on cell proliferation

**Figure 3:**
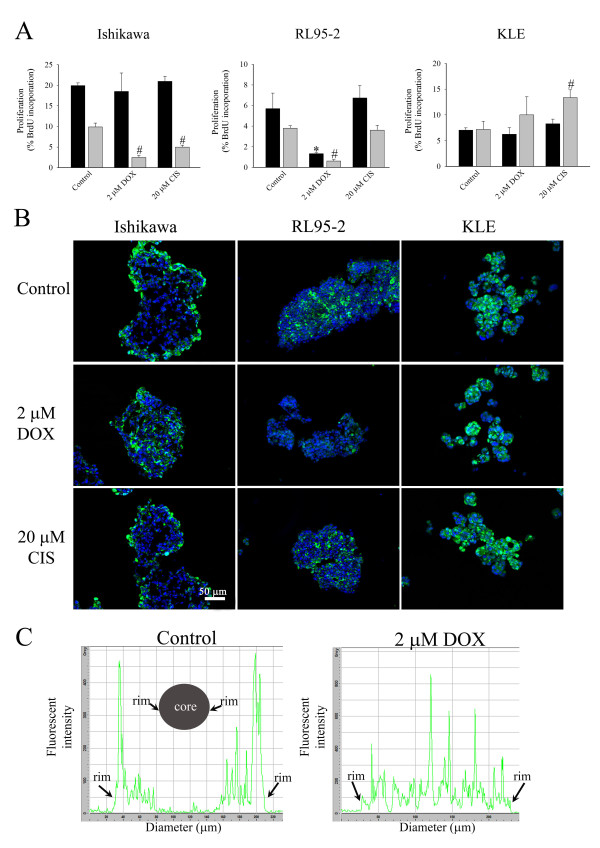
**The effect of doxorubicin (DOX) and cisplatin (CIS) on cell proliferation**. (A) Effects of doxorubicin and cisplatin on cell proliferation in multicellular structures and cell monolayers. After 48 hours treatment of cells with anticancer agents, BrdU was added to the cultures for 5 hours. Cells were trypsinised, fixed, permeabilised, exposed to anti-BrdU antibody, stained with anti-mouse conjugated to Alexa™ 488 nm and analysed with FACS. The (■) dark and (grey square symbol) grey bars represent multicellular structures and cell monolayers respectively. (*) *P *< 0.05, compared to control multicellular structures. (#) *P *< 0.05, compared to control cell monolayers. The data are presented as mean ± SEM from at least four separate experiments. (B) Effects on staining patterns of proliferating cell nuclear antigen (PCNA) by doxorubicin and cisplatin. Frozen sections of multicellular structures were cut, fixed and stained with anti-PCNA antibody. Ishikawa, RL95-2 and KLE 3D multicellular structures were studied. PCNA is stained green and the nucleus is blue. The scale bar is 50 μm. The images were representative of at least two separate experiments. (C) Fluorescent intensity profile of PCNA of control and DOX treated Ishikawa spheroids.

Noting that doxorubicin and cisplatin had less effect on apoptosis in 3D multicellular structures than cell monolayers, we hypothesised that these agents might, however, exert strong anti-proliferative effects. To investigate this, we used BrdU incorporation and staining of PCNA to analyse cell proliferation. Our results showed that cell proliferation of spheroids from Ishikawa cells was not affected by the anticancer drugs (Figure 3A). In contrast, proliferation of cell monolayers was significantly reduced by both drugs.

Doxorubicin reduced cell proliferation in both cell aggregates and cell monolayers of RL95-2 cells but cisplatin did not. Cell clusters of KLE cell line exhibited no change in cell proliferation after doxorubicin or cisplatin treatment. It is notable that in cell monolayers of KLE cell line proliferation was significantly increased after cisplatin treatment.

Immunohistochemistry of PCNA in frozen sections of 3D multicellular structures was employed to obtain details on changes in proliferation that might occur in limited regions. It was revealed that PCNA was localised at the rim of control spheroids of Ishikawa cell line (Figure 3B, C). After spheroids were treated with doxorubicin, the staining of PCNA was observed predominantly around the core of Ishikawa spheroids (Figure 3C). Cisplatin failed to increase the staining of PCNA in central regions in distinction from doxorubicin-treated spheroids. In contrast to spheroids of Ishikawa cells, the staining of PCNA in control cell aggregates of RL95-2 cells was predominantly localised adjacent to central regions. Doxorubicin substantially reduced the staining of PCNA. Treatment with cisplatin did not markedly alter the staining of PCNA. There was no observed change of PCNA staining in KLE cell clusters after treatment.

### Effects on upstream proliferative markers Erk1/2 and p-Erk1/2

**Figure 4:**
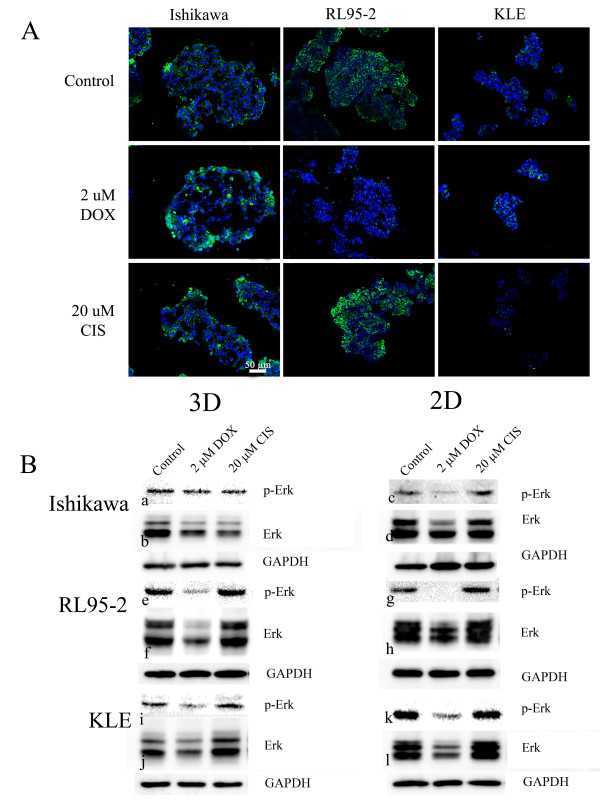
**Expression of Erk1/2 and p-Erk1/2**. (A) Effects on immunohistochemistry staining of active form of Erk (p-Erk) by doxorubicin and cisplatin. Multicellular structures of Ishikawa, RL95-2 and KLE cells were cut, fixed and stained with anti-p-Erk antibody. p-Erk was stained green and the nucleus is blue. The scale bar is 50 μm. (B) Western blotting of active form of Erk (p-Erk) and total expression of Erk. After 48 hours treatment with anticancer drugs, cells were lysed and proteins were separated and analysed by SDS-PAGE and Western blotting. The active form (p-Erk) and total expression of Erk from multicellular structures of Ishikawa (B:a-b,), RL95-2 (B:e-f), KLE (B:i-j) and cell monolayers of Ishikawa (B:c-d), RL95-2 (B:g-h), KLE (B:k-l).

**Figure 5:**
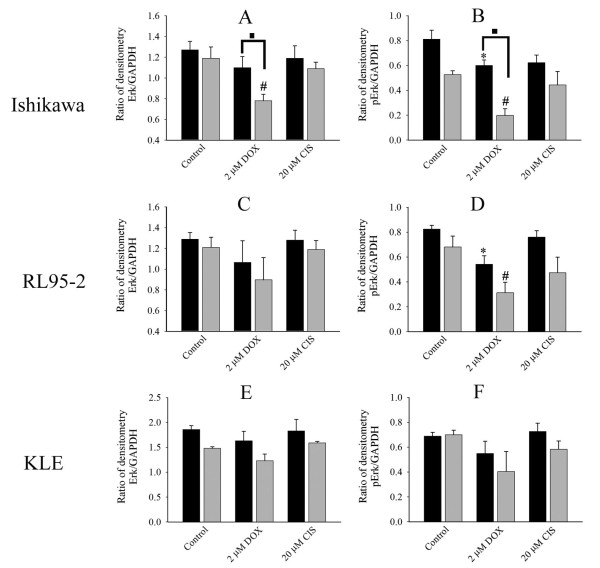
**Densitometry of Erk1/2 and p-Erk1/2**. Western blotting analysis of cell protein lysate was from 3D cell cultures and cell monolayers. GAPDH was used as control for loading. Densitometric analyses were performed using Alliance 4.7, Unitec (Cambridge, UK) and are presented as a ratio of Erk/GAPDH and pErk/GAPDH. The (■) dark and (grey square symbol) grey bars represent multicellular structures and cell monolayers respectively. (*) *P *< 0.05, compared to control multicellular structures. (#) *P *< 0.05, compared to control cell monolayers. (■) *P *< 0.05, comparison of between multicellular structures and cell monolayers. The data are presented as mean ± SEM from at least four separate experiments.

The effects of doxorubicin and cisplatin on cell proliferation may be a result of the inactivation of MAPK. To investigate this, immunohistochemistry of p-Erk1/2 in the multicellular structure of Ishikawa, RL95-2, and KLE cells was performed. Cells showed small vesicle-like staining at the rim and core regions (Figure 4A). However, after being treated with doxorubicin diffuse staining was observed in Ishikawa spheroids. Doxorubicin decreased the staining of p-Erk1/2 in cell aggregates of RL95-2 cells but did not alter the staining in KLE cell clusters. Cisplatin did not alter the observed staining of p-Erk1/2. We next evaluated the expression of total Erk1/2 and p-Erk1/2 by Western blotting. Spheroids of Ishikawa cells treated with doxorubicin did not significantly reduce the expression of total Erk1/2 (Figures 4B-b, 5A) but doxorubicin reduced p-Erk1/2 in spheroids of Ishikawa cells (Figure 5B). Furthermore, doxorubicin significantly reduced both Erk and p-Erk1/2 in cell monolayers of Ishikawa cells (Figures 4B-c, d, 5A, B). Interestingly, in doxorubicin treated Ishikawa cells levels of p-Erk1/2 in spheroids were maintained at substantially higher levels compared to cell monolayers (Figure 5B). The total Erk1/2 in cell aggregates and cell monolayers of RL95-2 cell line tended to reduce after doxorubicin treatment but the difference was not statistically significant (Figures 4B-f, 5h, 5C). However, p-Erk1/2 in both cell aggregates and monolayers of RL95-2 cells were significantly reduced after being treated with doxorubicin (Figure 4B-e, g, 5D). However, the level of p-Erk1/2 in cell aggregates was marginally higher than cell monolayer but it was not statistically significant. Doxorubicin also had a tendency (without statistical significance) to reduce total Erk and p-Erk1/2 in spheroids and cell monolayers of KLE cells (Figure 4B-j, i, l, k, 5E, F). Cisplatin had limited effects in multicellular structures and cell monolayers of all cell lines.

Therefore, alteration of cell proliferation may be associated with levels of phosphorylation of Erk1/2 but also it appears to be dependent on the individual cell line. The results suggest that 3D culture enhanced the levels of expression.

### Effects on Glucose metabolism

**Figure 6:**
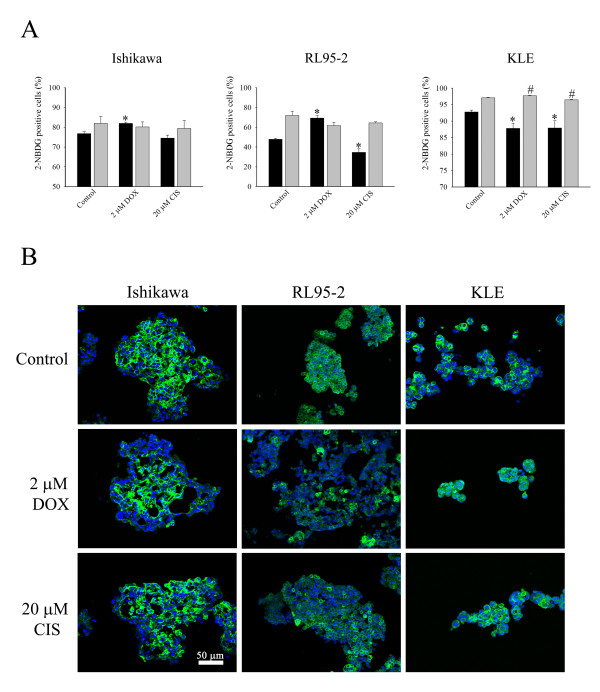
**The uptake of 2-NBDG and staining of Glut-1 in multicellular structures and cell monolayers**. (A) Effects on uptake of a fluorescent glucose analog 2-NBDG after cells were treated for 48 hours with doxorubicin and cisplatin. The (■) dark and (grey square symbol) grey bars represent multicellular structures and cell monolayers respectively. (*) *P *< 0.05, compared to control multicellular structures. (#) *P *< 0.05, compared to control cell monolayers. Data are expressed as the mean ± SEM from at least four independent experiments. (B) Effects on immunohistochemistry staining of Glut-1 by doxorubicin and cisplatin. Multicellular structures of Ishikawa, RL95-2, and KLE cells were cut, fixed and stained with anti-Glut-1 antibody. Glut-1 was stained green and the nucleus is blue. The scale bar is 50 μm.

Alteration of proliferation in 3D cell cultures and cell monolayers during drug treatment may also be associated with the increase of glucose metabolism in cancer cells. To test this hypothesis, we utilised the fluorescent glucose analogue, 2-NBDG, which enters cells via glucose transporter proteins (Gluts) including Glut-1. The results showed that the uptake of 2-NBDG was varied among cell lines (Figure 6A). KLE cells showed the highest activity of 2-NBDG uptake (cell monolayers, 97.04% ± 0.179, cell clusters, 92.75% ± 0.56), followed by Ishikawa cells (cell monolayers, 81.94% ± 3.64, spheroids, 76.76% ± 1.08) and RL95-2 cells (cell monolayers, 72% ± 4.2, cell aggregates, 47.87% ± 0.9). In addition, cell monolayers had greater uptake of 2-NBDG than cell clusters and aggregates in KLE and RL95-2 cell lines respectively (t-test, *P *< 0.05), but Ishikawa cell line did not show any difference between cell monolayers and spheroids.

Interestingly, after treatment with doxorubicin, the uptake of 2-NBDG in spheroids and cell aggregates of Ishikawa and RL95-2 cells, respectively, was increased whereas it was reduced in cell clusters of KLE cells (Figure 6A). However, there was no change in cell monolayers of Ishikawa and RL95-2 cells but there was an increase of 2-NBDG uptake in KLE cell monolayers. Cisplatin reduced the uptake of 2-NBDG in cell aggregates of RL95-2 cells and in both cell clusters and monolayers of KLE cells.

The increased uptake of 2-NBDG may be due to the upregulation of Glut -1 expression. To investigate this, we next examined immunofluorescent staining of Glut-1 protein. In the control spheroid of Ishikawa cells, the staining was observed predominantly in regions that were adjacent to the core but the staining was less at the rim of spheroids (Figure 6B). However, after the treatment with doxorubicin, strong staining was observed only at the core. Similarly, control cell aggregates of RL95-2 cells showed strong staining of Glut-1 at the rim and central region but the staining was reduced after doxorubicin treatment. Doxorubicin decreased plasma membrane-associated Glut-1 in KLE spheroids. Interestingly, in spite of cisplatin reducing the uptake of 2-NBDG by, staining of Glut-1 was not markedly altered in RL95-2 aggregates and KLE cell clusters.

Therefore, the effects on proliferation by doxorubicin and cisplatin were not clearly associated with alteration of glucose metabolism and that was confirmed by the pattern of uptake of 2-NBDG and expression of Glut-1. In addition, the level of glucose metabolism was not readily associated with the expression of Glut-1.

### Effects on endogenous antioxidant protein by drug treatment

**Figure 7:**
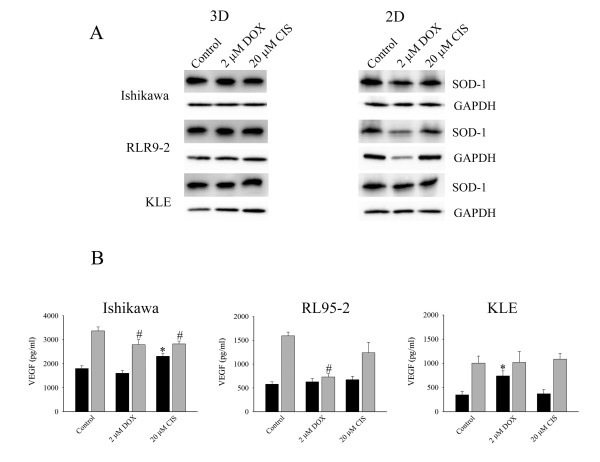
**Expression of superoxide dismutase-1 (SOD-1) and effects on secretion of vascular endothelial growth factor (VEGF) from multicellular structures and cells monolayers**. (A) Expression of SOD-1 of cells cultured in 3D and 2D cell models. SOD-1 was maintained at high levels in cancer cells cultured as 3D multicellular structures compared with 2D cell monolayers. (B) Secreted VEGF in the media was collected and examined by ELISA. The (■) dark and (grey square symbol) grey bars represent 3D cell cultures and cell monolayers respectively. (*) *P *< 0.05, compared to control multicellular structures. (#) *P *< 0.05, compared to control cell monolayers. Data are expressed as the mean ± SEM.

The insensitivity of tumours to cytotoxic agents may be associated with the elevated expression of endogenous antioxidant proteins in cancer cells. To examine the protective role of these antioxidant proteins during drug exposure in 3D and 2D cell cultures, we selected superoxide dismutase-1 (SOD-1) as a surrogate marker for antioxidant proteins. All cell lines cultured in 3D cell structures expressed high levels of SOD-1 and its expression was maintained after the exposure to doxorubicin and cisplatin (Figure 7A). Cell monolayers of Ishikawa and RL95-2 cell lines decreased SOD-1 expression after treatment with both drugs. The level of SOD-1 expression in cell monolayers of KLE did not change.

### Effects on secretion of VEGF

Increasing tumourigenic activity is often associated with elevated secretion of VEGF. Next, we asked whether doxorubicin and cisplatin inhibits secretion of VEGF. Therefore, VEGF secreted by 3D cell cultures and cell monolayers were examined. Cells from 3D cell cultures generally secreted less VEGF than cell monolayers (Figure 7B). Spheroids of Ishikawa and cell aggregates of RL95-2 cells did not change VEGF secretion after doxorubicin treatment but it was significantly decreased in cell monolayers of these cell lines (Figure 7B). Doxorubicin, paradoxically, increased VEGF release from cell clusters, but not cell monolayers of KLE cells. Cisplatin also increased VEGF secretion from spheroids of Ishikawa cells, but it reduced secretion from monolayers. Cisplatin had no detectable effects on VEGF release from RL95-2 or KLE cells.

Our results suggested doxorubicin and cisplatin selectively altered secretion of VEGF in a manner, which was dependent on cancer cell line and was also cell culture method dependent.

### Effects on p(Ser473)-Akt after drug treatment

**Figure 8:**
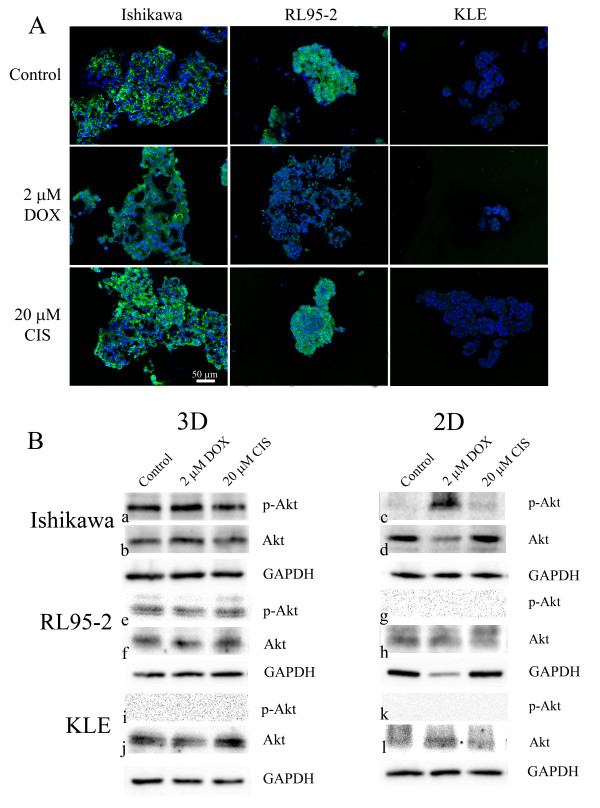
**Expression of Akt and p-Akt**. (A) Effects on immunohistochemistry staining of p-Akt by doxorubicin and cisplatin. Multicellular structures of Ishikawa, RL95-2 and KLE cells were cut, fixed and stained with anti-p-Akt antibody. p-Akt was stained green and the nucleus is blue. The scale bar is 50 μm. (B) Western blotting of active form Akt (p-Akt) and total expression of Akt. After 48 hours treatment with anticancer drugs, cells were lysed and proteins were separated and analysed by SDS-PAGE and Western blotting. The active form (p-Akt) and total expression of Akt of multicellular structures of Ishikawa (B:a-b,), RL95-2 (B:e-f), KLE (B:i-j) and cell monolayers of Ishikawa (B:c-d), RL95-2 (B:g-h), KLE (B:k-l).

Upregulation of p-Akt may enhance tumour progression and mediate resistance to drugs [18]. Strong staining of p-Akt was observed at the cell membrane in the control of Ishikawa spheroids and RL95-2 cell aggregates (Figure 8A). Doxorubicin-treated spheroids exhibited less p-Akt associated membrane staining but increased diffuse staining in the cytoplasm. Similar results were observed in doxorubicin-treated RL95-2 cell aggregates. Cisplatin did not induce obvious changes. On the other hand, cell clusters of KLE cells showed weak staining of p-Akt.

Western blotting showed that spheroids of Ishikawa cells expressed p-Akt, which was not altered by anticancer drugs (Figure 8B-a). Cell monolayers of Ishikawa cells had low expression of p-Akt (Figure 8B-c) and doxorubicin slightly increased p-Akt expression (Figure 8B-c). Cell aggregates of RL95-2 cell line (Figure 8B-e) expressed p-Akt, which was not altered by anticancer drugs. Interestingly, cell monolayers of RL95-2 cell line had no detectable levels of p-Akt (Figure 8B-g) even though there were substantial levels of total Akt (Figure 8B-h). Neither cell aggregates nor cell monolayers of KLE expressed detectable p-Akt.

The results are consistent with the notion that the constitutive expression of p-Akt may enhance resistance to doxorubicin and cisplatin in 3D multicellular structures of Ishikawa and RL95-2 cells.

## Discussion

The microenvironment of multicellular structures regulates gene and protein expressions, which are distinct from those in cell monolayer counterparts [10,19]. However, the use of multicellular structures in investigations of responses to drugs is novel and not widespread, and never previously studied in endometrial cancer. Thus, there is a gap in data as it pertains to the study of endometrial cancer. To the best of our knowledge, this is the first study on the use of multicellular structures in endometrial cancer and it further investigates the antitumour potential of clinical drugs. We have considered the potential of cell responses in an *in vitro *3D cell model to provide useful prognostic biomarkers that may have beneficial clinical relevance.

In our conditions, distinct multicellular morphologies of cancer cell lines were observed as compact spheroids (Ishikawa cell line), cell aggregates (RL95-2 cell line), and cell clusters (KLE cell line). The exact mechanism, which may influence the spheroid formation, is still poorly defined but there are few studies that note the potential relationships of individual cancer phenotypes, production of extracellular matrix (ECM) and the expression of integrin subunits. For instance, the formation of compact spheroids in ovarian cancer cells may be associated with production of ECM, displaying a mesenchymal phenotype, and influence the invasive behaviour of cancer cell lines [20]. Small quantity of basement membrane extract (Matrigel) added to cell aggregations can stimulate cell aggregates to form compact spheroids [21], thereby suggesting the contribution of ECM in the early stage of compact spheroids formation. Possibly promotion of rapid cell aggregation is induced by integrin-ECM in the initial stage of spheroid assembly [12,22]. The development from loose aggregates to compact spheroids may also be dependent on cell adhesion protein, E-cadherin [22]. Cell lines used in our investigations express measurable levels of β1 integrin subunit and Ecadherin [23-25]. Therefore, these adhesion molecules may be not directly involved in the early steps of spheroid formation. It is possible that these cell lines may produce various degrees of ECM, which may facilitate the initial cell-cell and cell-ECM interactions that generate compact spheroids. In the present study, we did not investigate the molecular nature of the ECM within spheroids and it remains to be determined in the conditions of the current study.

Cell aggregates and clusters derived from RL95-2 and KLE cell lines respectively, contained fewer apoptotic cells after doxorubicin treatment compared to their cell monolayers. However, apoptosis was also increased in Ishikawa cells but there was no difference between spheroids and cell monolayers. This led us to speculate that the compactness of spheroids in Ishikawa cells plays only a minor role in protection of cells from apoptosis after doxorubicin treatment. We had confirmed diffusion of fluorescent doxorubicin to the central region of spheroids, indicating that the limitation of drug accessibility was not responsible for insensitivity to doxorubicin in this study, although spheroids greater than 250 μm in diameters may have reduced doxorubicin penetration [15].

The mutation of *TP53 *tumour suppressor gene is common in endometrial cancer. Cancer cells carrying wild-type p53 protein can exert a variety of anti-proliferative effects, including induction of apoptosis [26]. Accordingly, loss of wild-type p53 functions could lead to increased chemoresistance. Endometrial cancer cell lines used in our study all have *TP53 *gene alterations; deletion in RL95-2, and mutations in KLE and Ishikawa [27,28] cell lines. However, the sensitivity to doxorubicin was markedly different between 3D multicellular structures and cell monolayers of RL95-2 and KLE cell lines. Our observations may suggest that expression and functionality of p53 protein may be distinct in 3D cultures compared to cell monolayers.

There are many possible explanations for multicellular structures showing greater resistance to doxorubicin than cell monolayers. One possibility is that a number of cancer cells at the central core of spheroids are in a quiescent state, in which DNA topoisomerase II levels are low. As a consequence, the number of doxorubicin-induced DNA strand breaks is lower than in fast growing cells [6]. This is consistent with our data showing that PCNA containing cells in RL95-2 cell aggregates were observed at core regions and they were more sensitive to doxorubicin than Ishikawa spheroids. Second, spheroid formation is a process, in which cancer cells survive by anchorage independent pathways that is a hallmark of cancer metastasis. Data suggests survival and resistance to anticancer drugs by anchorage-independent pathways are sustained by an activation of growth factor related signalling pathways [29], which are differently modulated in the distinct microenvironments.

It is interesting that cisplatin did not induce apoptosis or necrosis in our present study. Others have shown that cisplatin reduced cell proliferation and increased apoptosis in cell monolayers of Ishikawa and KLE cell lines. These discrepancies may be due to the use of different techniques to analyse effects of the drug [30].

The difference of activity of doxorubicin and cisplatin in inducing apoptosis in 3D multicellular structures and cell monolayers led us to investigate cell proliferation. Cell proliferation of Ishikawa spheroids was unchanged after doxorubicin treatment. Surprisingly, more proliferative cells were observed in the central region after treatment. This demonstrated that different cell population became proliferative in different regions of spheroids. These observations indicate that there is a heterogeneous cell population in spheroids [31]. It is also possible that spheroids after drug-treatment may have altered cell-cell interaction at the rim, which enabled increased penetration of nutrition to the inner regions of spheroids, thereby initiating cell proliferation of quiescent cells [11,32]. This phenomenon has been reported in tumours of patients after they received chemotherapy-radiation [33,34], which suggests the 3D model may provide interactions that induce cancer cells to behave similarly to an *in vivo *environment.

Cell proliferation appears to be linked with p-Erk1/2 [35]. The association of increased expression of p-Erk with acquisition of spheroid resistance to chemotherapeutic drugs supported this idea. Both cell aggregates and monolayers of RL95-2 cells reduced p-Erk after doxorubicin treatment and subsequently decreased cell proliferation. However, the reduction of p-Erk in spheroids of Ishikawa cells did not parallel proliferation, which was unaffected by the treatment. Therefore, Erk in compact spheroids of Ishikawa cells and cell aggregations of RL95-2 cells may activate distinct pathways to regulate cell proliferation. In contrast to Ishikawa and RL95-2 cells, cell clusters of KLE treated with doxorubicin did not exhibit reduced p-Erk and cell proliferation. Taken together, this may suggest that each cell line has various pathways to regulate cell proliferation and that such pathways may be adapted to the microenvironments of tumours.

The results also showed there was lack of correlation of glucose metabolism in cell proliferation with apoptotic events after drug treatments, supporting previous observations [36]. Doxorubicin increased glucose metabolism in Ishikawa cell spheroids and RL-952 cell aggregates but it decreased glucose metabolism in KLE cell clusters. In contrast, cisplatin decreased glucose metabolism in RL-952 and KLE 3D cell cultures. The results may suggest the distinct responses of glucose metabolism to anticancer agents depending on cancer cell lines. In our study, staining of Glut-1 was observed at the plasma membrane of cells and was also adjacent to the core of the spheroids. Strikingly, after treatment with doxorubicin, the staining of Glut-1 was mainly in the central region and was localised in the cytoplasm of cells. The reduction of Glut-1 staining, however, did not correlate with the increase of glucose metabolism with doxorubicin treatment. Furthermore, it was surprising that cell monolayers of Ishikawa and RL95-2 cell lines did not alter the uptake of 2-NBDG after treatment. Also, it is noted that doxorubicin and cisplatin have different effects on the uptake of 2-NBDG, which may suggest that drugs have specific targets that are distinct in each cancer cell line. It is possible that many Gluts, besides Glut-1, may be responsible for the uptake of 2-NBDG [37]. Alternatively, the activity of Glut-1 rather than the expression of protein may be responsible for the increase of uptake 2-NBDG.

The observed resistance to anticancer drugs could also be due to upregulation of endogenous antioxidant proteins. Doxorubicin and cisplatin have been shown to increase ROS, which is believed to be the primary mechanism contributing to the induction of apoptosis in cancer cells [38,39]. Our findings suggest that SOD-1, which is localised mainly in the cytoplasm of cancer cells, may protect cells from cytotoxic insult. However, it seems likely that multicellular structures produce a high level of SOD-1 compared with the cell monolayers, in agreement with others [40,41]. This led us to speculate that nutrient depletion in the 3D multicellular morphology may generate cellular metabolic stresses, which in turn increase the production of endogenous antioxidant molecules in a homeostatic response. Thus, the microenvironment within multicellular structures can significantly impact on the success of chemotherapeutic treatments.

It is well known that secretion of VEGF is strongly stimulated by tumour hypoxia. Increase of HIF-1α expression in a 3D spheroid has been demonstrated [42,43]. However, there are many inconsistent data regarding the association VEGF and hypoxic microenvironment in the 3D spheroid. VEGF localisation was strongly observed in the outer cell layers that were directly exposed to the growth medium in spite of having the low oxygen level in the core of spheroids [14]. Increased secretion of VEGF is evidenced in colorectal cancer spheroids but this is not affected by hypoxia [16]. The relatively short culture period in our experiments (5 days) and small size of multicellular morphology could however explain the difference from independent reports. In our study, multicellular structures produced less VEGF compared to cell monolayers. This finding may suggest that there are other factors in addition to the influence of hypoxia that can contribute to elevated levels of VEGF production and secretion. Interestingly, doxorubicin and cisplatin had no reductive effects on VEGF secretion in multicellular structures but instead exhibited selective stimulatory effects. This has important clinical implications in that the angiogenic and growth enhancing activities of VEGF are paradoxically encouraged by the putative anticancer drugs in 3D tissue microenvironments. The current finding may suggest that the effects of anticancer agents on VEGF activity may be as a result of the different molecular pathways according to individual characteristics of the tumours [14,44].

The immunostaining showed that spheroids of Ishikawa and cell aggregates of RL95-2 cells constitutively expressed p-Akt. It is known that Ishikawa and RL95-2 cells harbour PTEN mutated inactive protein [45], and that leads to the upregulation of the Akt signalling pathway. Nevertheless, there was less p-Akt expressed in cell monolayers than spheroids. Therefore, our data may suggest that microenvironments within spheroids, such as EGFR-related pathways [46], are able to produce intracellular cues to trigger and sustain p-Akt activation. Interestingly, p-Akt in cell monolayers of Ishikawa was up-regulated after exposure to doxorubicin. This result implies that increased p-Akt levels are a potential defensive mechanism [47]. Some differences between spheroids and monolayers have been ascribed to PI3K/Akt/mTOR activities [48]. Further, our results also revealed that KLE cells did not have readily detectable p-Akt staining, consistent with previous reports that grade 3 tumours had wild type PTEN [49] and low levels of p-Akt [30]. Therefore, the resistance to doxorubicin in cell clusters of KLE may be modulated by Akt independent pathways. Alternatively, constitutive activation may be reduced in cell monolayers and less compact spheroids [50,51] as it noted in KLE cell line.

We report the pathways that are altered by anti-cancer drugs in a 3D multicellular structure are dependent on oncogenic genotype, thus adding to the burgeoning literature that cautions against ignoring individual responsiveness in clinical situations. This study undertook a comparison between characteristics of cancer cells in monolayers and 3D multicellular structures and thereby providing direct evidence of the influence of the cellular microenvironment. For the first time such information is available for endometrial cancer. In this study, there appears to be no significant effects in cisplatin-treated spheroids. Of particular note was the observation that anti-cancer drugs might increase VEGF secretion.

## Conclusion

Our investigations demonstrated that there were variations in metabolic activities, growth pattern, response to chemotherapy among cancer cell lines, and cell culture methods. In general, the intracellular mediators in 3D multicellular morphologies demonstrated greater resistance to chemotherapy than in monolayers. These observations have important implications with regard to the *in vitro *study of anticancer treatments for endometrial cancer. In addition, a chemotherapeutic sensitivity assay in a 3D cell model that supports culture of primary cancer cells from patients may offer a closer approximation of clinical sensitivity than a monolayer culture and may also enable the tailoring of individual chemotherapy treatments in patients with advanced endometrial cancer.

## Competing interests

The authors declare that they have no competing interests.

## Authors' contributions

KC performed all *in vitro *studies and participated in preparation of the manuscript. PS and JE participated in experimental designs and assisted the preparation of manuscript. All authors have read and approved the final manuscript.
